# Transcriptomic Profiling of *Paulownia fortunei* (Seem.) Hemsl. Roots in Response to Chromium and Copper Stress

**DOI:** 10.3390/genes16050595

**Published:** 2025-05-18

**Authors:** Jiang Su, Xinfeng Pan, Kanghua Xian, Chuanming Fu, Jinxiang He, Baojun Liu, Jinhan Sang, Ningzhen Huang

**Affiliations:** 1 Guangxi Key Laboratory of Plant Conservation and Restoration Ecology in Karst Terrain, Guangxi Institute of Botany, Guangxi Zhuang Autonomous Region and Chinese Academy of Sciences, Guilin 541006, China; su3374766861@163.com (J.S.); panxinfeng@gxib.cn (X.P.); xkh@gxib.cn (K.X.); fuchuanming@gxib.cn (C.F.); hjx@gxib.cn (J.H.); bjliumail@126.com (B.L.); a13196221635@163.com (J.S.); 2 Ministry of Education Key Laboratory for Transboundary Ecosecurity of Southwest China, Yunnan Key Laboratory of Plant Reproductive Adaptation and Evolutionary Ecology, Institute of Biodiversity, School of Ecology and Environmental Science, Yunnan University, Kunming 650504, China

**Keywords:** *Paulownia fortunei*, heavy metal pollution, transcriptome analysis, differential gene expression, soil remediation

## Abstract

Background: Soil heavy metal pollution by chromium (Cr) and copper (Cu) is a global environmental concern. Methods: This study evaluated Cr/Cu accumulation in *Paulownia fortunei* tissues and analyzed its root transcriptome under Cr and Cu stress to elucidate molecular response mechanisms. Results: Findings revealed significantly higher Cr and Cu accumulation capacity in roots compared to stems and leaves. Transcriptome sequencing identified 6017 and 2265 differentially expressed genes (DEGs) under Cr and Cu stress, respectively. These DEGs were primarily involved in redox reactions, stress responses, transcriptional regulation, transmembrane transport, and metabolism. Quantitative PCR of 20 selected genes validated dynamic expression changes under stress. Weighted Gene Co-expression Network Analysis (WGCNA) identified distinct co-expression modules associated with Cr and Cu. Hub gene analysis implicated *Pfo_020668* and *Pfo_019190* in Cr response, while *Pfo_010312* and *Pfo_000197* may enhance Cu tolerance via cell wall polysaccharide synthesis regulation. Pathways related to pyruvate metabolism and proteasome were significantly enriched under Cr stress, whereas amino acid metabolism pathways were prominent under Cu stress. Conclusions: Differentially expressed transporter genes suggest potential roles in heavy metal uptake and transport. This transcriptomic analysis provides novel insights into *P. fortunei’s* molecular responses to Cr and Cu stress, offering a foundation for utilizing this species in soil phytoremediation efforts.

## 1. Introduction

Soil heavy metal contamination is a challenging environmental problem worldwide, with copper (Cu) and chromium (Cr) being the most significant metal contaminants. These contaminants originate primarily from industrial activities, mining, smelting, metal processing, chemical production, pesticide use, and vehicle exhaust emissions [1,2]. Chromium in soil primarily exists in two forms: trivalent chromium (Cr (III)) and hexavalent chromium (Cr (VI)) [3]. Trivalent chromium (Cr (III)), also known as “bioactive chromium” or “beneficial chromium”, is generally considered an essential trace element for humans and plants. It plays a role in insulin regulation of blood sugar levels. In soil, Cr (III) is relatively stable, less mobile, and has lower bioavailability [4]. Cr (VI) is a toxic and carcinogenic substance that poses significant risks to human health and the environment. It has high solubility and mobility, making it easy to migrate from soil into groundwater and surface water. Cr (VI) can accumulate through the food chain, threatening ecosystems and human health [5,6]. Studies indicate that 11% of contaminated sites in the United States are polluted with Cr, while 14% of contaminated sites in Japan are affected by Cr pollution and the area of Cr-contaminated soil accounts for over 5% of the total area of polluted soils in China [7,8]. Cu is a trace element that plays a crucial role in the growth and development of both plants and animals. However, it is also a significant heavy metal pollutant found in contaminated soils. In certain regions worldwide, Cu levels have been observed to exceed the soil’s natural carrying capacity [9]. The global problem of Cu contamination in soil is serious; it has been reported that the Cu content in uncontaminated soils typically ranges from 3.8 to 93.8 mg/kg, while in highly contaminated areas, such as copper mining regions, Cu concentrations can reach as high as 104 to 6841 mg/kg [10]. Maintaining the sustainable usability of soil is contingent upon the control and elimination of sources of soil pollution. This necessitates the regulation of both the quantity and rate of heavy metal generation. Ensuring that these metals degrade and stabilize in soil without leading to significant accumulation that causes pollution is fundamental for preventing heavy metal contamination in soils.

Currently, commonly used soil remediation techniques include physical remediation technologies, chemical remediation technologies, and bioremediation technologies [11]. Physical remediation technologies tend to have high operational costs and can cause considerable environmental damage. They are generally suitable for small areas or soils with low permeability but may not be applicable for larger or highly permeable soils [12,13]. Chemical remediation technologies involve the use of chemical stabilizers or leaching agents, which may introduce new chemical substances into the soil, potentially leading to secondary pollution. Additionally, chemical treatments can alter soil pH, organic matter content, and other critical soil properties, thereby affecting soil fertility and crop growth. Certain chemical remediation methods may also negatively impact soil microorganisms and other biological activities [14,15]. Conversely, bioremediation circumvents the concerns that accompany physical and chemical remediation methods. This technology employs natural biological processes to diminish the concentration of heavy metals in soils [16,17]. Dou et al. [18] successfully isolated and acclimatized a specific microbial strain, CT13, which exhibits high tolerance to acid rain and cadmium (Cd). Khan et al. [19] identified and isolated a total of three metal-tolerant fungal strains from heavy metal-contaminated soils: M1DGR, M3Ai, and M2Ai, with M3Ai demonstrating significant potential for soil remediation of Cd and Cr. In addition to microorganisms, plants also serve as effective remediation systems. Research has shown that *Rhazya stricta* Decne possesses strong absorption capabilities for lead Pb and Cu, making it suitable for phytoremediation in mildly contaminated soils in arid regions [20].

*P. fortunei*, commonly known as the white flower paulownia, plays a significant role in the remediation of heavy metals in soil. Wang et al. [21] found that mature *P. fortunei* exhibits high tolerance to Pb and zinc (Zn), while seedlings tend to absorb Cu more readily.Studies on *P. fortunei* seedlings have demonstrated that manganese (Mn) accumulates primarily in the roots, with a translocation factor (defined as the ratio of Mn concentration in shoots to roots, [Mn]shoot/[Mn]root) less than 1.00, indicating limited translocation of Mn to aboveground parts [22]. Rational utilization of soil may enhance the accumulation of copper by plants, thereby facilitating ecological restoration of copper mine tailings. Furthermore, the addition of mushroom residue has been shown to improve the phytoremediation potential of *P. fortunei* for manganese slag and its capacity for Pb and Zn remediation by increasing the binding capacity of cell walls and vacuoles, thereby reducing heavy metal toxicity effects on *P. fortunei* [22].

Research on the metal accumulation capacity and soil remediation potential of *P. fortunei* has predominantly focused on physiological levels, with a paucity of in-depth analysis at the molecular level regarding how this species responds to heavy metal stress. The genomic responses and molecular mechanisms of *P. fortunei* under heavy metal stress are not yet fully understood. The present study therefore aims to sequence the transcriptome of *P. fortunei* seedlings under Cu and Cr stress, in order to identify differentially expressed genes (DEGs) and further construct gene co-expression networks associated with response mechanisms at the molecular level. The findings of this study may enhance our understanding of the potential Cu and Cr tolerance processes in *P. fortunei* seedlings and provide a molecular biological basis for future studies on the mechanisms of heavy metal remediation in contaminated soils using this species.

## 2. Materials and Methods

### 2.1. Plant Materials and Treatment

The source and cultivation method for *P. fortunei* seedlings is consistent with that described by [23], and the seedlings with the same growth were treated with 250 mg/L Cr (K_2_Cr_2_O_7_, 2405 μM) and 40 mg/L Cu (CuSO_4_·5H_2_O, 630 μM) respectively. Plants were harvested and separated into stem, leaf, and root for the measurement of dry weight after three days. Cr contents were estimated according to Gill et al. [24] and for Cu contents, the methodology of Li et al. [25] was followed with some modifications.

### 2.2. Total RNA Extraction, Reliability Assessment and RNA Sequence Analyses

The RNAprep Pure Kit (DP432, Tiangen Biotech, Beijing, China) with a gDNA-removal step was used to isolate the total RNA. The RNA integrity was assessed by 1.2% agarose gel electrophoresis, while the RNA yield and purity were determined with a NanoDrop 2000 spectrometer (Thermo Fisher Scientific, Waltham, MA, USA). After the extraction of total RNA, the eukaryotic mRNA was enriched using Oligo(dT) beads. The enriched mRNA was fragmented into short fragments using a fragmentation buffer and reverse transcribed into cDNA. The purified double-stranded cDNA fragments were end-repaired, followed by the addition of A base and ligation to Illumina sequencing adapters. The ligation products were purified using AMPure XP Beads. Finally, the cDNA library was obtained by PCR enrichment.

### 2.3. Sequencing Data Analysis

Use script code to process raw reads in fastq format. Clean reads were obtained after removing the raw reads containing adaptors and polyN and low-quality reads. Q20 and Q30 [26], GC content and sequence repeat level of clean reads were calculated. Hisat2 (v2.1.1) [27] software was used to align high-quality clean reads to reference genome ‘*P. fortunei*’(GCA_019321725.1).

### 2.4. Differential Expression Analysis, Functional Annotation and Enrichment Analysis

FPKM (Fragments Per Kilobase of transcript per Million fragments mapped) was used for genome-wide and transcriptome-based key gene mining and functional studies to show gene expression levels. Differential expression analysis between sample groups was performed using DESeq2 software (v1.48.1) [28], and FDR < 0.01 and FC ≥ 2 were used as thresholds to determine differentially expressed genes.

Functional annotations were performed in Nr, COG, Swiss-Prot and eggNOG databases using BLAST (v2.14.0) (E value ≤ 1 × 10^−5^). GO and KEGG enrichment analysis was performed using R software (v4.4.2) package ‘GOseq’ [29] and KOBAS software (v3.0) [30]. The threshold of *p* < 0.05 was used to determine the significant enrichment of GO functional items and KEGG pathways by differential genes.

### 2.5. Weighted Gene Co-Expression Network Analysis

Weighted gene co-expression network analysis (WGCNA) (v1.0) was used to analyze the differentially expressed genes [31]. The genes with FPKM ≥ 1 are used for module construction, Fold = 0.5 to control the similarity of Module fusion, minModuleSize = 30, and the remaining parameters are defaulted, and the module is obtained by using the automatic network construction function

### 2.6. Quantitative Real-Time Polymerase Chain Reaction

To validate the transcriptome sequencing data and gain a deeper understanding of the gene expression profiles in *P. fortunei* under Cr (250 mg/L) and Cu (40 mg/L) stress, a quantitative real-time PCR (qPCR) analysis was conducted on 20 selected genes over a time course from 0 to 48 h post-treatment. The primers were designed with Primer Premier 5.0 software and embodied in (Table S1). The *tubulin 1* gene was selected as the reference gene [23]. For each treatment, 1 μg of total RNA was reverse-transcribed into cDNA (20 μL) using the PrimeScript^TM^ RT Master Mix (RR036Q, Takara Biotech, Nanning, China). Subsequently, these cDNAs were diluted tenfold and used as templates for qPCR reaction. qPCR assays were conducted according to Su et al. [23], The PCR reaction (20 μL) comprised of 2 × TB Green Premix Ex Taq (10 μL), 10 × dilution cDNA (1 μL), 10 μMol/L forward/reverse primers (0.8 μL), 50 × Rox Reference Dye (0.4 μL), and ddH_2_O (7 μL). The following amplification conditions were used: 95 °C for 20 s; 40 cycles of 95 °C for 10 s and 58 °C for 30 s. The melting curve was determined at a range of 60–95 °C. All assays were repeated 3 times. Negative controls using total RNA or ddH2O instead of cDNA for all samples were applied. The relative expression level of each gene was calculated by 2^−∆∆Ct^ method.

### 2.7. Data Processing

Microsoft Excel 2019 and Origin 2024 software were used for data analysis and graphing.

## 3. Results

### 3.1. Accumulation of Cr and Cu in Different Tissue Types of Paulownia Seedlings

**Figure 1:**
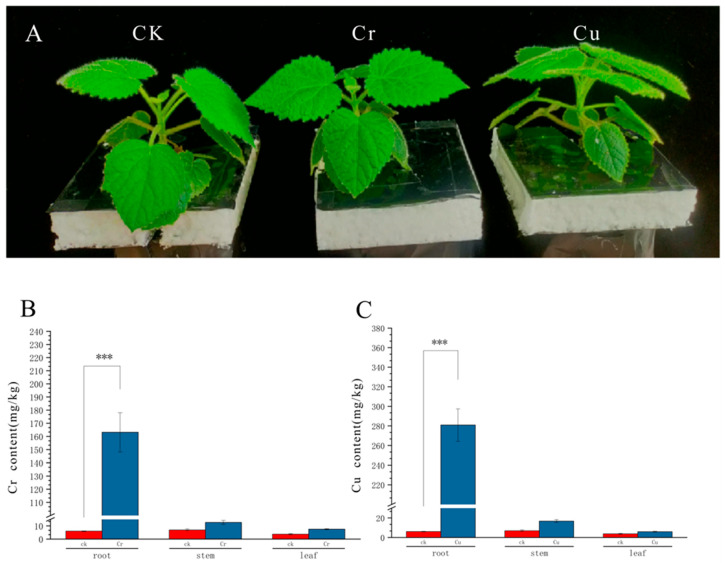
Enrichment of Cr and Cu in roots, stems and leaves of *P. fortunei*; (**A**) Phenotypes after three days of treatment with Cr (250 mg/L) and Cu (40 mg/L) concentrations; (**B**) After three days of Cr (250 mg/L) concentration treatment, the enrichment content of Cr in roots, stems and leaves was compared with CK; (**C**) After three days of Cu (40 mg/L) concentration treatment, the enrichment content of Cu in roots, stems and leaves was compared with CK. Values and error bars represent the means and standard errors of four replications. CK, control; *** indicates significant differences at *p* < 0.001 level; Error bars = mean ± standard error (SE; *n* = 3).

Paulownia seedlings were cultured for three days in Cu, Cr and untreated control groups respectively (Figure 1A). Subsequently, root, stem, and leaf samples were collected to assess heavy metal accumulation and were compared with the control group (CK). In the Cr treatment, the Cr content in roots increased significantly compared to CK (*p* < 0.001) and was substantially higher than levels in stems and leaves (Figure 1B, Table S2). While Cr levels also trended higher in stems and leaves compared to CK, these increases were less pronounced. Similarly, for Cu treatment, Cu concentration in roots showed a significant increase compared to CK (*p* < 0.001) and was markedly higher than in stems and leaves (Figure 1C, Table S2). Stem and leaf Cu levels also showed an increase compared to CK, but this was not statistically significant.

### 3.2. RNA-Seq Data Quality and Statistics

**Figure 2:**
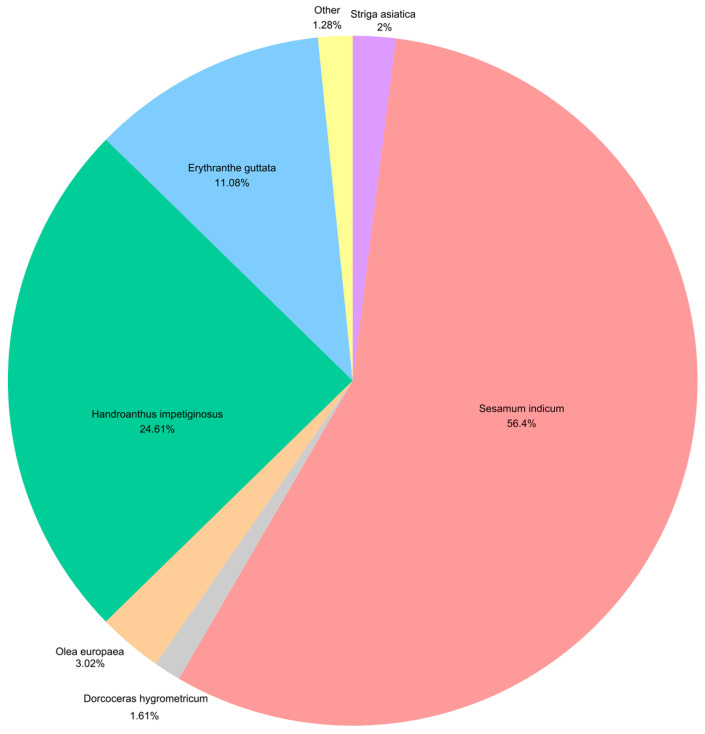
Distribution of NR-annotated species.

Transcriptome sequencing of *Paulownia fortunei* (Scrophulariaceae, Lamiales) roots under Cr and Cu stress for 24 h generated 79.56 Gb of clean data, with each sample exceeding 6.06 Gb and a Q30 base percentage of 92.79%. De novo assembly yielded 31,836 unigenes. Among these, 56.40% (17,955 unigenes) were annotated in *Sesamum indicum* L. (Pedaliaceae, Lamiales), 24.61% (7836 unigenes) in *Handroanthus impetiginosus* (Mart. ex DC.) Mattos (Bignoniaceae, Lamiales), 11.08% (3526 unigenes) in *Erythranthe guttata* (DC.) G.L. Nesom (Phrymaceae, Lamiales), 3.02% (961 unigenes) in *Olea europaea* L. (Oleaceae, Lamiales), 2% (638 unigenes) in *Striga asiatica* (L.) Kuntze (Orobanchaceae, Lamiales), and 1.61% (514 unigenes) in *Dorcoceras hygrometricum* Bunge (Gesneriaceae, Lamiales). These six species, all within the order Lamiales, exhibited the highest sequence similarity to *P. fortunei* unigenes, reflecting their close phylogenetic relationships (Figure 2).

### 3.3. Differentially Expressed Genes 

**Figure 3:**
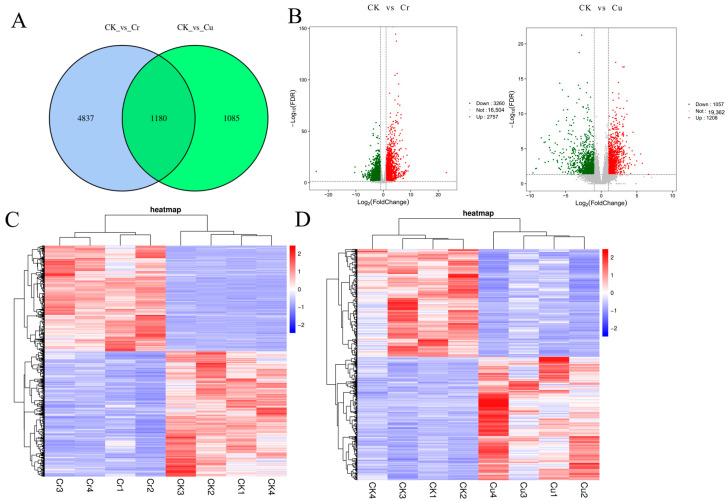
Differentially expressed genes in paulownia roots from plants exposed to either Cr or Cu for 24 h compared to unexposed plants. (**A**) Venn diagram of differentially expressed genes that were unique to and shared by plants exposed to Cr or Cu. (**B**) The volcano map illustrates the number of upregulated and downregulated genes; (**C**) The expression genes after Cr treatment; (**D**) The expression genes after Cu treatment.

A total of 6017 and 2265 DEGs were identified in the comparisons of CK vs. Cr and CK vs. Cu, respectively (Figure 3A). Among these, 1180 DEGs were shared between the CK vs. Cr and CK vs. Cu groups, while 4837 DEGs were specific to the CK vs. Cr group (representing genes specifically responsive to Cr stress), and 1085 DEGs were unique to the CK vs. Cu group (genes specifically responsive to Cu stress) (Figure 3A). Under Cr stress, 2757 DEGs were upregulated, while 3260 DEGs were downregulated. In contrast, under Cu stress, 1208 DEGs were upregulated and 1057 DEGs were downregulated (Figure 3B).

### 3.4. GO and KEGG Analysis of Differentially Expressed Genes

**Figure 4:**
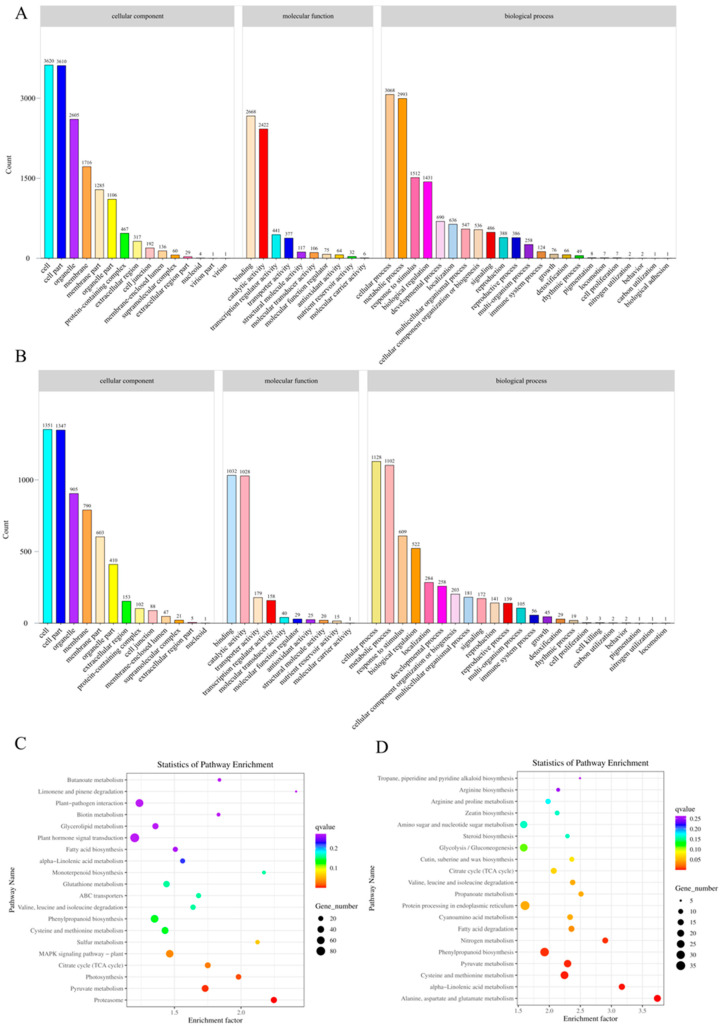
GO and KEGG for Cr and Cu. (**A**) Gene ontology (GO) enrichment classification map of differentially expressed genes under Cr Stress. (**B**) GO enrichment classification map of differentially expressed genes under Cu Stress. (**C**) Kyoto encyclopedia of genes and genomes (KEGG) enrichment analysis of differential expression genes under Cr Stress. (**D**) KEGG enrichment analysis of differential expression genes under Cr Stress.

Gene Ontology annotation analysis showed that most DEGs regulated by Cr and Cu were annotated as “cellular process” or “metabolic process” in biological process (BP) ontology and as “cell”, “cell part”, or “organelle part” in cellular component (CC) ontology, and “binding”, “catalytic activity” in molecular function (Figure 4A,B). KEGG annotation analysis showed that DEGs regulated by Cr were annotated as “Proteasome”, “Pyruvate”, “Photosynthesis” (Figure 4C). KEGG annotation analysis showed that DEGs regulated by Cu were annotated as “Alanine, aspartate and glutamate metabolism”, “α-Linolenic acid metabolism”, “Cysteine and methionine metabolism” (Figure 4D).

### 3.5. Expression Profiles of Putative Transporters Response to Cr and Cu

**Figure 5:**
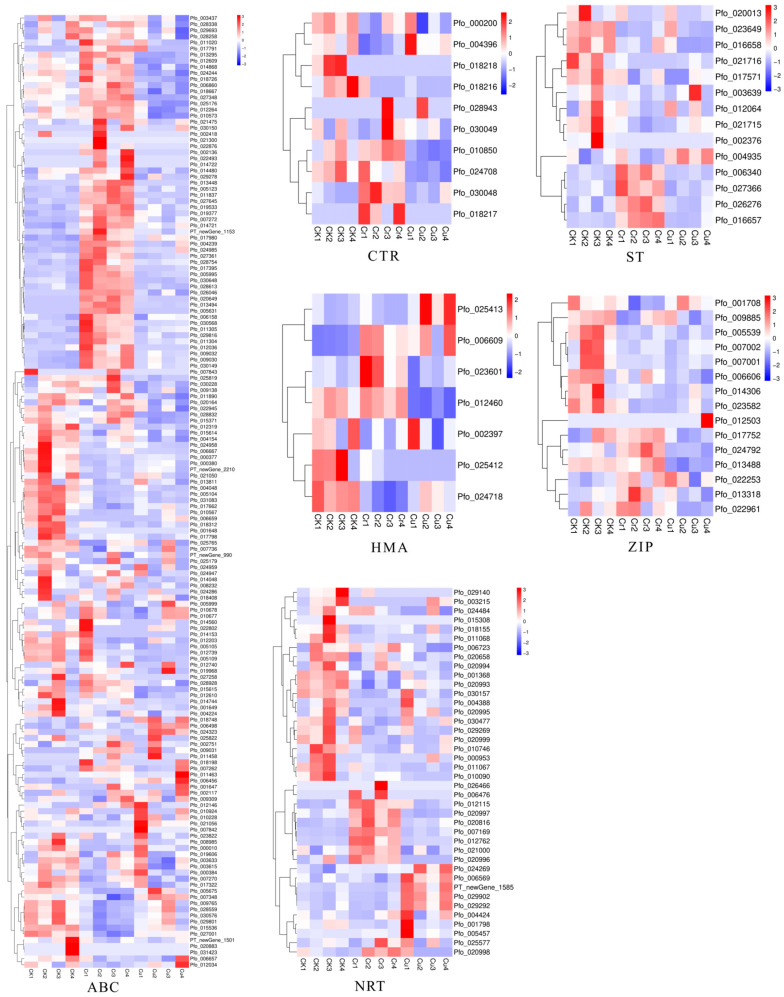
Heat maps of gene expression levels for six major transporter families related to metal ions transport after Cr and Cu treatment.

According to the transcriptome data, we found that a large number of transporter-related genes were involved in the transfer processes of Cr and Cu uptake, a number of other genes encoding transporters were identified. These genes were classified into several families: the ATP-binding cassette (ABC) family (157 genes), zinc/iron regulated transporter protein (ZIP) family (15 members), nitrate transporter protein (NRT) family (40 genes), heavy metal ATPase (HMA) family (7 members), copper transporter (CTR) family (10 members), and sulfate transporter (ST) family (14 members) (Figure 5, Table S3). The observed differential expression among these transporter families (Figure 5) strongly indicates their involvement in the process of absorbing and transporting Cr and Cu ions.

### 3.6. Co-Expression Network Analysis

**Figure 6:**
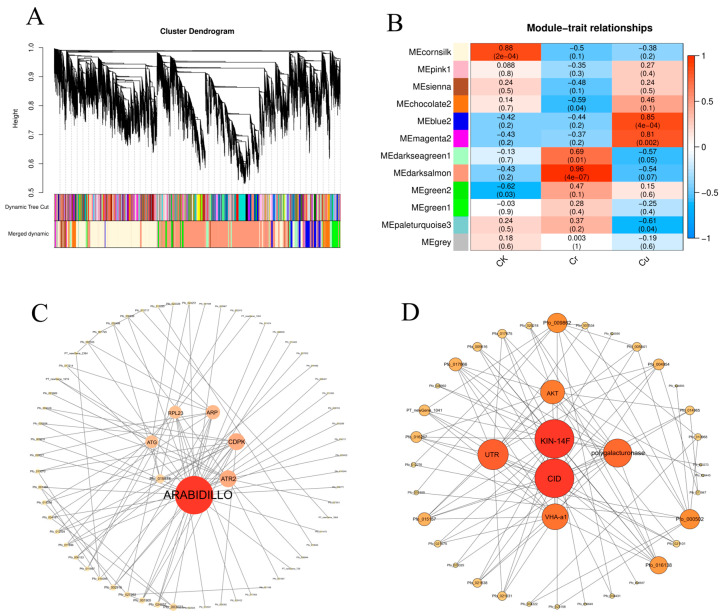
Establishment of a network of weighted gene co-expression and correlation analysis. (**A**) Gene clustering tree displaying 12 co-expression modules, distinguished by different merged colors in the lower panel. (**B**) Module-trait correlations represented by a color scale ranging from blue (−1) to red (1). (**C**) The darksalmon module is visualized based on the connectivity between genes. (**D**) The blue2 module is visualized based on the connectivity between genes.

Based on pairwise correlations and gene expression trends across all samples, coexpression networks were constructed using the normalized expression data of 22,800 genes from 12 samples (four biological replicates for CK, Cr, and Cu). A total of 12 modules were generated, with each color representing a specific module that contains a cluster of highly correlated genes (Figure 6A). Notably, four modules exhibited significant relationships with Cr and Cu stress. The “darksalmon” and “darkseagreen1” modules primarily displayed expression specificity related to Cr, while the “blue2” and “magenta2” modules were mainly associated with Cu (Figure 6B). KEGG enrichment analysis of the blue2 module revealed that genes were enriched in pathways such as “Protein processing in endoplasmic reticulum”, “Chaperones and folding catalysts”, “Membrane trafficking”, and “Propanoate metabolism” (Figure 6C). In contrast, genes in the magenta2 module were enriched in pathways including “Translation”, “Ribosome”, “Proteasome”, “Ribosome biogenesis”, “Citrate cycle (TCA cycle)”, and “Messenger RNA biogenesis” (Figure 6D). For the darksalmon module, the top 100 genes based on connectivity were visualized, revealing that *Pfo_030353* (ARABIDILLO, Armadillo/β-catenin-like repeat) had the highest degree, followed by *Pfo_019190* (ATR, NADPH-dependent diflavin oxidoreductase), *Pfo_020668* (CDPK, Calcium-dependent protein kinase), *Pfo_015345* (ARP, Actin-related protein) and *Pfo_020836* (ATG, Autophagy-related protein) (Figure 6C). Similarly, in the blue2 module, visualization of the top 100 genes based on connectivity showed that *Pfo_027922* (CID, Polyadenylate-binding protein-interacting protein) had the highest degree, followed by *Pfo_014537* (KIN-14F, Kinesin-like protein), *Pfo_010312* (UTR, UDP-galactose/UDP-glucose transporter), *Pfo_000197* (polygalacturonase, Probable polygalacturonase), *Pfo_007173* (VHA-a1, V-type proton ATPase subunit a1), and *Pfo_028080* (AKT, Probable potassium channel)(Figure 6D).

### 3.7. Quantitative Real-Time PCR Analysis of Selected Genes Under Cr and Cu Stress

**Figure 7:**
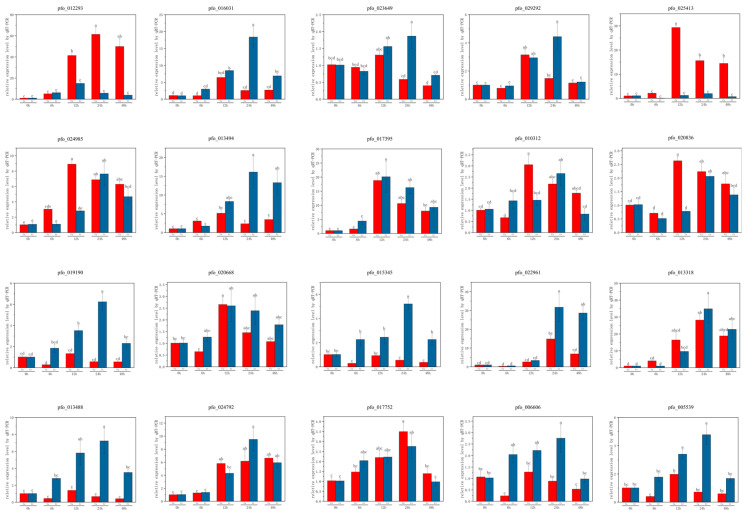
Temporal expression patterns of 20 selected genes in *P. fortunei* under Cr and Cu stress determined by qPCR. Different letters indicate significant difference according to Duncan’s multiple range test at a significance level of *p* < 0.05.

To validate the transcriptome sequencing data and investigate gene expression dynamics in *P. fortunei* under Cr (250 mg/L) and Cu (40 mg/L) stress, quantitative real-time PCR (qPCR) was performed on 20 selected genes over a 0–48-h time course (Figure 7).

#### 3.7.1. Genes Associated with Detoxification and Stress Response

Detoxification-related genes exhibited distinct regulation under heavy metal stress. *Pfo_012293* (CYP71A1), encoding Cytochrome P450 71A1, showed a marked increase under Cr stress, peaking at 24 h and remaining elevated through 48 h, with moderate upregulation under Cu stress, implying a role in oxidative detoxification. *Pfo_023649* (SULTR3), a probable sulfate transporter, displayed a biphasic response under Cr stress—upregulation at 6–12 h followed by a decline—while remaining unaffected under Cu stress, linking it to Cr-specific sulfur metabolism. *Pfo_029292* (NPF8.1), an NRT1/PTR family member, was significantly regulated under Cr stress but showed negligible changes under Cu stress, suggesting Cr-specific transport functions. *Pfo_020836* (ATG16), an autophagy-related protein, increased gradually under Cu stress, indicating a detoxification role via autophagy.

#### 3.7.2. Genes Related to Signaling and Structural Integrity

Genes tied to signaling and structural integrity also responded dynamically. *Pfo_020668* (CDPK10), a calcium-dependent protein kinase, showed strong upregulation under Cu stress, peaking at 12–24 h, with moderate Cr stress responses, underscoring its signaling role. *Pfo_016031* (FAF4), encoding Protein FANTASTIC FOUR 4, increased gradually under Cr stress, potentially aiding stress-adaptive signaling, with minimal Cu stress changes. *Pfo_015345* (ARP), an actin-related protein, was significantly downregulated under Cu stress from 6–48 h, suggesting cytoskeletal adjustments, while showing slight Cr stress fluctuations. Comparative analysis revealed overlapping yet distinct responses: *Pfo_012293* (CYP71A1) and *Pfo_017395* (ABCB4) were upregulated under both stresses, with stronger Cr and Cu responses, respectively, while *Pfo_023649* (SULTR3) and *Pfo_029292* (NPF8.1) were Cr-specific, and *Pfo_010312* (UTR4) and *Pfo_020836* (ATG16) were Cu-responsive. These qPCR findings confirm transcriptome data and highlight temporal expression dynamics, aiding identification of key genes for enhancing *P. fortunei’*s phytoremediation potential in heavy metal-contaminated soils.

## 4. Discussion

### 4.1. Heavy Metal Tolerance and Remediation Potential of P. fortunei

In practical soil remediation scenarios, mixed pollution by multiple heavy metals is often encountered. As an important potential plant for soil bioremediation, it is essential for species to demonstrate significant accumulation capabilities for various heavy metals. To identify suitable plants for the remediation of heavy metal-contaminated soils, studies have been conducted on different plant types to assess their effectiveness in accumulating multiple heavy metals. For instance, *Phragmites australis* (Cav.) Trin. ex Steud exhibits differential translocation capacities for multi-metal contaminants (Cu, Pb, As, Zn), highlighting its adaptability to polymetallic pollution scenarios [32]. Moreover, aquatic plants such as *Typha latifol* L. (Typhaceae) and *Thelypteris palustris* Schott (Thelypteridaceae) demonstrate time-dependent enhancement in zinc and copper enrichment efficiencies in wastewater systems, coupled with sustained biomass growth, underscoring their viability for aquatic heavy metal remediation [33]. Comparative analyses of fiber crops reveal that *Hibiscus cannabinus* L. (Malvaceae) achieves significantly higher remediation efficiencies for Cr, Co, and Cd than *Linum usitatissimum* L. (Linaceae), while the latter shows preferential manganese removal capabilities. Notably, dual-season cropping strategies further amplify their combined remediation performance [34]. Our results confirm that *P. fortunei* employs a root-based accumulation strategy for Cr and Cu (Figure 1), consistent with findings for Pb, Zn, and Cd by Zhang et al. [35]. Zhang et al. also demonstrated that translocation factors (TF) for heavy metals in *P. fortunei* are typically less than 1, indicating limited transport to aerial parts. This strategy, focusing on phytostabilization within the root system rather than phytoextraction, is common among metal-tolerant woody plants and minimizes damage to photosynthetic tissues. These hyperaccumulator plants hold significant value for practical applications in the remediation of contaminated land, serving as potential bioremediation materials with promising prospects for the removal of heavy metal pollutants from soil.

According to the results of this study, *P. fortunei* effectively utilized its root system to accumulate the maximum amount of heavy metals, thereby reducing their translocation to aerial parts and enhancing its tolerance to heavy metals. This finding is consistent with the results reported by [36]. These observations indicate that *P. fortunei* possesses unique and effective heavy metal tolerance, making it an ideal resource for soil remediation. Therefore, investigating the physiological and molecular mechanisms underlying *P. fortunei*’s tolerance to heavy metals holds significant practical and scientific importance.

Compared with other species, *P. fortunei* shows a higher tolerance to Cr and Cu. For example, compared with Cr-sensitive rice (*Oryza sativa*) [37], the roots of *P. fortunei* accumulated more Cr (Figure 1B) and showed a unique response pattern at the transcriptome level. Such as the significant enrichment of the “Pyruvate metabolism” and “Proteasome” pathways (Figure 4C). These pathways may help *P. fortunei* cope with Cr-induced oxidative stress and protein damage by eliminating reactive oxygen species (ROS) and degrading damaged proteins. In addition, the high accumulation ability of *P. fortunei*’s roots may be a key characteristic of its tolerance, reducing toxic effects by restricting the transport of heavy metals to the above-ground parts, which is consistent with the strategy of tolerant plants reported in the literature such as *Phragmites australis* [32]. In contrast, sensitive species such as rice exhibited more significant growth inhibition and oxidative damage under Cr stress [37], suggesting that the molecular response of *P. fortunei* might be more effective. In the future, these mechanisms can be further verified by comparing with the transcriptomes of sensitive and closely related species.

### 4.2. Differentially Expressed Genes in Response to Cr and Cu

Transcriptomics has been established as a powerful tool for studying gene expression in non-model plants at the molecular level. In this study, a total of 32,968 transcripts were obtained after trimming and assembly, which surpasses previous assemblies of *P. fortunei* [38] (Figure S1). Overall, 6017 differentially expressed genes (DEGs) were identified in *P. fortunei* under Cr treatment, while 2265 DEGs were identified under Cu treatment. These results indicate significant changes in gene expression and suggest that *P. fortunei* is more sensitive to Cr stress compared to Cu stress.

KEGG and GO analyses of DEGs under Cr treatment revealed that the induced and repressed genes are associated with redox potential control, various stress responses, transcriptional regulation, transmembrane transport, signal transduction, and biosynthesis and metabolism (Table 1). Notably, “Pyruvate metabolism” (map00620) and “Proteasome” (map03050) emerged as significantly enriched KEGG pathways in this study. Cr stress leads to the production of ROS, which can damage cellular components. This has been demonstrated in studies involving *Oryza sativa* and the macrofungus *Pleurotus ostreatus* [37,39]. Pyruvate, known for its antioxidant properties, can scavenge ROS and help mitigate oxidative stress [40]. The accumulation of ROS can trigger oxidative damage to proteins, resulting in an increased expression of proteasome subunits to meet the enhanced demand for protein degradation. Additionally, pathways such as “Sulfur metabolism” (map00920) and “Glutathione metabolism” (map00480) were also enriched. The thiol groups in glutathione (GSH) have a unique ability to form stable thiol-metal bonds, coupled with high solubility, making GSH effective in detoxifying various heavy metals [41]. For Cr, high concentrations of GSH can rapidly form unstable Cr (VI)-GSH complexes that subsequently decompose slowly [42]. Furthermore, the presence of Cr-GSH complexes in vacuoles can alleviate Cr-induced cellular damage, thereby enhancing the stress tolerance of *P. fortunei*.

KEGG and GO analyses of the differentially expressed genes (DEGs) under Cu treatment revealed functional enrichments similar to those observed with Cr treatment. However, the DEGs in the Cu treatment were specifically enriched in pathways related to “Alanine, aspartate and glutamate metabolism” (map00250), “α-Linolenic acid metabolism” (map00592), and “Cysteine and methionine metabolism” (map00270). Copper induces oxidative stress, including lipid peroxidation reactions within plant tissues, which can severely damage cellular membranes [43,44]. Glutamate and methionine serve as precursors for GSH, an important antioxidant. Nagalakshmi et al. found that as Cu concentrations increased, the protein thiol content in Scenedesmus cells rose, while GSH levels significantly decreased, increasing the activity of enzymes such as γ-glutamylcysteine synthetase, glutathione S-transferase, and glutathione peroxidase, while reducing the activity of glutathione reductase, leading to alterations in GSH homeostasis [45]. Tahjib et al. demonstrated that GSH inhibits copper uptake in rice, enhancing antioxidant activity and mitigating copper toxicity [46]. In Brassica napus, GSH interacts with copper to form complexes with thiol groups, thereby improving resistance to copper stress [47]. Younis et al. found that under copper stress, the GSH content in *Phaseolus vulgaris* L. increased, resulting in enhanced copper tolerance [48].

A key aspect of heavy metal tolerance is subcellular compartmentalization [35]. Studies on *P. fortunei* have shown that metals like Pb, Zn, Cu, and Cd are primarily sequestered within the cell wall and the vacuole (soluble fraction). Our transcriptomic data aligns with this mechanism. For instance, the WGCNA identified hub gene *Pfo_010312* (UDP-galactose/UDP-glucose transporter) and *Pfo_000197* (polygalacturonase) in a Cu-associated module, potentially contributing to cell wall polysaccharide synthesis and modification, thereby enhancing metal binding capacity in the apoplast, a major sequestration site. Furthermore, the differential expression of various transporter families, particularly ABC and HMA pumps (Figure 5, Table S3), likely facilitates the transport of Cr and Cu across the tonoplast for sequestration within the vacuole, effectively removing them from the cytoplasm.This emphasis on root sequestration and compartmentalization contrasts with the high sensitivity reported for young *P. fortunei* seedlings to Cu stress [21], suggesting that these tolerance mechanisms are more robust or fully developed in more mature plants or specifically in root tissues compared to seedlings.

### 4.3. Transporter Protein

The maintenance of heavy metal homeostasis in plants is a tightly regulated process, and numerous genes involved in heavy metal responses have been identified in current research. Plants enhance their tolerance to heavy metals by transporting metal ions to vacuoles or other organelles. In the alga *Scenedesmus acutus* (Scenedesmaceae, Sphaeropleales), plasma membrane sulfate transporters (SULTRs) play a role in Cr tolerance. Upregulation of these transporters, particularly high-affinity ones, is proposed to limit Cr (VI) uptake due to competitive inhibition, as the transporters preferentially bind sulfate (SO_4_^2−^) over the chemically similar toxic chromate anion (CrO_4_^2−^). Concurrently, the enhanced sulfate uptake resulting from this upregulation boosts the synthesis of sulfur-containing defense compounds, further contributing to tolerance [49]. The higher accumulation of Cr in maize roots may lead to the upregulation of ABC transporter G family member 29, which is targeted by miR444f. This establishes an ABC-miRNA regulatory network in response to Cr stress [50]. Similarly, the current study results indicate that several members of the ABC transporter family are also highly expressed in *P. fortunei* under Cr stress. Therefore, it is inferred that these ABC family members play a significant role in the regulation of Cr ion accumulation. In the CTR, the COPT1 transporter is responsible for copper uptake and is primarily located in the plasma membrane of root tips. When copper is deficient, plants upregulate the expression of COPT1 to acquire significant amounts of copper from the growth substrate [51]. The absorption of copper by plants generates hydroxyl radicals (OH^−^), which bind to non-selective cationic channels (NSCC) in the plasma membrane, opening calcium channels and promoting root growth. COPT2 expression is also induced under copper deficiency, with further increases observed in conditions of both copper and iron deficiency [52]. In this study, multiple CTR family genes exhibited differential expression under Cu treatment, suggesting similar functions to their homologs in *Arabidopsis*. Additionally, several CTR genes showed differential expression under Cr treatment, with similar results reported for *Miscanthus sinensis* under Cr stress [53]. This leads to the hypothesis that CTR family genes regulate the response pathways to Cr stress or that Cr stress affects the transport function of Cu ions in plants. Therefore, further functional characterization of these genes is warranted. The NRT (Nitrate Transporter) family primarily facilitates the absorption and transport of nitrates within plants; however, studies indicate that they may also play roles in the uptake and transport of other ions, such as Cr and Cu. In cucumber, at Cu levels higher than 20 µM, plants demonstrated either strongly reduced or abolished NO^3−^ uptake activity. Nevertheless, the transcriptional modulation of both the nitrate transporter CsNRT2.1 and the accessory protein CsNRT3.1 was not coherent with the hindered NO^3−^ uptake activity [54]. In Iris, genes related to soil nitrogen assimilation (NRT, nrtA, and nrtC) were upregulated under Cr stress [55]. The relevant NRT genes in this study may also serve similar functions.

### 4.4. Co-Expression Network

In this study, based on WGCNA analysis and the KEGG database, four distinct co-expression modules were identified that respond to Cu and Cr stress. Notably, the darksalmon module showed the highest correlation with Cr, while the magenta2 module was most closely associated with Cu. KEGG enrichment analysis of the genes in the darksalmon module revealed significant pathways, including “Ribosome”, “Proteasome”, “Citrate cycle (TCA cycle)”, and “Oxidative phosphorylation”. The genes in the blue2 module were enriched in “Protein processing in endoplasmic reticulum” and “Propanoate metabolism”. Subsequently, hub genes were selected based on top degree connectivity. *Pfo_020668* from the darksalmon module was identified as a CDPK gene (calcium-dependent protein kinase). Previous studies have indicated that signaling pathways within plant cells are activated under stress conditions, with calcium ions (Ca^2+^) acting as key second messengers involved in regulating plant responses [56,57]. *Pfo_019190* was identified as a flavodoxin (Fld), which is known to accept electrons from NAD(P)H. This suggests that AtTAH18, a homologous gene, is a flavoprotein capable of electron transfer. Therefore, it is hypothesized that Cr stress may activate extracellular reductase activity to catalyze electron transfer for heavy metal detoxification. In submerged plants like *Callitriche cophocarpa*, exposure to hexavalent chromium induced a new NAD(P)H-dependent dehydrogenase, FQR1, which is considered a detoxification protein that helps protect cells from oxidative damage. FQR1 exhibits quinone reductase activity and can catalyze the transfer of two electrons from NAD(P)H to various substrates, potentially including hexavalent chromium [58].

In the Blue2 module, genes *Pfo_010312* and *Pfo_000197* are associated with the metabolic pathway of UDP-galactose synthesis. Galactose (Gal) is a major component of cell wall polymers, glycolipids, and glycoproteins, with UDP-galactose (UDP-Gal) serving as its primary metabolite. UDP-Gal plays a crucial role in the biosynthesis of cell wall components and is involved in various biological processes, including the synthesis of glycoproteins and glycolipids. Understanding the function of these genes will enhance our knowledge of UDP-Gal’s significance in plant physiology and its potential applications [59]. Polysaccharide groups on the cell wall, such as carboxyl, aldehyde, amino, and phosphate groups, can participate in the immobilization of heavy metal ions. These negatively charged functional groups can interact with metal cations through various reactions, thereby sequestering them within the cell wall and reducing their entry into the cytoplasm [60]. Research by [61] indicates that uronic acid residues play a significant role in the adsorption of heavy metals by the cell walls of *Sargassum* spp. Konno et al. [62] found that the accumulation of copper in the fern *Lygodium prothallium* is primarily associated with the pectic galacturonic acid polysaccharides in the cell wall. Hemicellulose components in castor bean roots not only accumulate a substantial amount of copper in the cell wall but also exhibit the fastest response to copper stress [63].

## 5. Conclusions

Transcriptome sequencing analysis has provided a wealth of transcriptomic information on *P. fortunei* under Cu and Cr stress conditions. Subsequent bioinformatics approaches have identified a set of differentially expressed genes (DEGs) responsive to Cu and Cr stress; however, further research is needed to clone these genes, validate their functions, and investigate their specific regulatory mechanisms in *P. fortunei*. This study further demonstrates the high accumulation capacity of *P. fortunei* for Cu and Cr, suggesting that future field trials should focus on its potential for remediating soils contaminated with these heavy metals. According to the experiment, the trunk does not have a strong ability to accumulate Cr and Cu, but its biomass is relatively large, and it may also have a repair effect, but further experiments are needed to verify it. In addition to evaluating the extraction efficiency and remediation period for Cu and Cr, it is essential to analyze how the plant interacts with other soil elements, particularly major nutrient elements, during the remediation process.

## Figures and Tables

**Table 1:** Summary of Major Differences in Transcriptomic Response of *P. fortunei* Roots to Chromium (Cr) and Copper (Cu) Stress.

Aspect of Response	Cr Stress Response	Cu Stress Response
Total DEGs	6017	2265
Unique DEGs	4837	1085
Key Enriched Pathways (KEGG)	Proteasome, Pyruvate metabolism, Sulfur metabolism, Glutathione metabolism	Alanine, Aspartate, Glutamate metabolism, α-Linolenic acid metabolism, Cysteine/Methionine metabolism
Prominent Molecular Mechanisms	Significant involvement of protein degradation, energy metabolism, and sulfur/glutathione pathways.	Stronger emphasis on specific amino acid metabolism precursors for GSH and potential membrane/cell wall related responses.
Transporter Families/Genes	Members from ABC, NRT, CTR, ST families showed differential expression; specific genes varied.	Members from ABC, ZIP, NRT, HMA, CTR, ST families showed differential expression; specific genes varied.
Representative Top Hub Genes	*Pfo_030353*(ARABIDILLO), *Pfo_019190* (ATR/Flavodoxin), *Pfo_020668* (CDPK)	*Pfo_027922*(CID), *Pfo_014537* (KIN-14F), *Pfo_010312*(UDP-Gal transporter), *Pfo_000197* (polygalacturonase)

## Data Availability

All data are included in the article and its Supplementary Materials.

## Supplementary Materials

The following supporting information can be downloaded at: https://www.mdpi.com/article/10.3390/genes16050595/s1, Figure S1: Number of transcripts. Table S1: qPCR primer information for the 20 selected genes; Table S2: The concentration of Cr and Cu in the roots, stems, and leaves of *paulownia fortunei;* Table S3: Potential transporter gene information in response to Cr and Cu stress. The project metadata is archived in https://www.ncbi.nlm.nih.gov/sra/PRJNA1242429 (accessed on 27 march 2025).
