# Supplementary figures and images for: Transcriptomic Profiling of Paulownia fortunei (Seem.) Hemsl. Roots in Response to Chromium and Copper Stress
Source: Genes (Basel). 2025 May 18;16(5):595. doi: 10.3390/genes16050595 (PMC12110757; doi:10.3390/genes16050595)

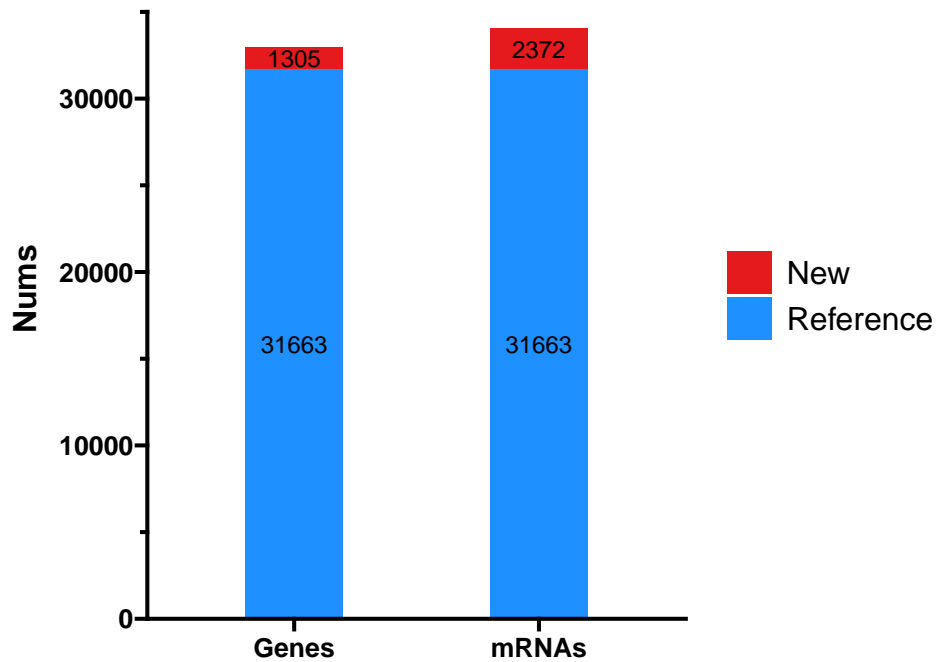

Supplement: Supplementary file 1 [file genes-16-00595-s001.zip › FIG S1.pdf]
